# Exploring the Effectiveness of the Optimized Activity Autonomous Choice Program in Day Care Center

**DOI:** 10.1002/alz70858_100704

**Published:** 2025-12-25

**Authors:** Yi‐Ting Ting, Pei‐Chin Pan, Hsiu‐Ling Lin, Huei‐Ling Huang

**Affiliations:** ^1^ Chang Gung University of Science and Technology, Taoyuan, Taiwan; ^2^ A Holistic Care Service Center for Long‐Term Care, Sisters of Providence‐Miracle Home, New Taipei, Taiwan

## Abstract

Taiwan will enter a super‐aged society by 2025. To promote aging in place, the government is actively promoting the setting up a day care center for the elderly in every junior high school district to delay aging and reduce the family caregiver's burden. Day care services have become one of the most highly utilized community‐based long‐term care services. In Taiwan, about 70% of the clients served by day care centers are persons with dementia, and individuals can maintain better functioning through participation in activities.

Studies have shown that autonomy is highly correlated with quality of life, especially for older adults. When individuals have the autonomy to choose the activities they want to participate in, they tend to be more engaged and derive more satisfaction. The project aims to identify factors contributing to low participation rates and propose targeted improvements through an optimized activity autonomous choice program.

This project was implemented in a day care center in North Taiwan, which served 57 clients, primarily elderly individuals, with a significant portion diagnosed with dementia. Despite general satisfaction with daily activities, the rate of autonomous activity selection remains low. Phase 1 of the project conducted questionnaires and interviews to gather insights into the challenges faced by clients, particularly those with cognitive impairments. Factors affecting the autonomy of choice were identified, including the routineness of activity arrangements, communication barriers and reduced ability of comprehension due to dementia. In response to the influencing factors, we have developed an “optimized activity autonomous choice program”.

After 8 weeks of intervention, the results showed significant improvements: satisfaction with activity arrangements rose from 80% to 88%, and the ability to choose activities independently increased from 30% to 100%. Additionally, the perceived availability of suitable activities improved markedly.

In conclusion, the optimized activity autonomous choice program successfully enhanced client satisfaction and autonomy in participation, aligning with previous findings that emphasize fulfilling basic psychological needs as crucial for improving the quality of life in older adults. Future recommendations include ongoing assessment of client preferences and diversifying activity options to further stimulate engagement and motivation.

